# Bilateral distal transradial access for subclavian artery stenosis intervention

**DOI:** 10.3389/fcvm.2025.1634574

**Published:** 2025-10-15

**Authors:** Lin Chen, Xiaofang Chen, Mingchen Sun, Toe Wai Wai Naing, Zhaokai Li, Min Lai, Zixin Tian, Ye Cheng, Huiyuan Kang, Yan Wang

**Affiliations:** Xiamen Cardiovascular Hospital of Xiamen University, School of Medicine, Fujian Branch of National Clinical Research Center for Cardiovascular Diseases, Xiamen, China

**Keywords:** subclavian stenosis, subclavian stent, bilateral distal transradial access, novel method, radial artery occlusion

## Abstract

**Background:**

Conventionally, subclavian stenting has been performed via the femoral artery, but this approach is associated with complications such as bleeding, patient discomfort, and prolonged recovery. The transradial artery access (TRA) has gained popularity due to its lower vascular complication rates, though it carries a risk of radial artery occlusion (RAO). The distal transradial access (dTRA) has emerged as an alternative with lower occlusion rates.

**Objective:**

This study aimed to assess the safety and technical feasibility of bilateral distal transradial access for percutaneous interventions in subclavian artery stenosis.

**Methods:**

We present 10 cases of subclavian artery stenosis, in whom diagnostic angiography was performed by SIM2 catheter via the contralateral side access. Ipsilateral side distal radial artery was subsequently accessed for balloon-expandable stents delivery.

**Results:**

Among the 10 patients, 9 were male and 1 was female, with a mean age of 65.8 years. The systolic pressure difference between the left and right arms was 21.00 mmHg. Among them, 2 cases had 80% stenosis, 2 cases had total occlusive lesions, and 6 cases had 90% stenosis. 1 case had right subclavian artery stenosis, while 9 cases had left subclavian artery stenosis. The results showed a 100% success rate for bilateral distal radial artery puncture and a 100% completion rate for subclavian stenosis intervention procedures. No RAO or subclavian artery restenosis was observed during the 1-month follow-up.

**Conclusion:**

In patients with subclavian stenosis bilateral dTRA is a safe and minimally invasive method for patients and ergonomically comfortable for operators.

## Introduction

Subclavian artery stenosis is not uncommon and can be found in approximately 2% of general and 7% in clinical population (1). Subclavian artery stenosis compromises blood flow to the brain and arm, and can steal blood from an internal mammary artery graft, causing stroke risk, limb symptoms, or recurrent cardiac ischemia after CABG (2). Conventionally, subclavian stenting has been successfully performed via femoral and brachial access for many years. Although transfemoral access(TFA) offers advantages such as delivery of larger devices and reduced spasm, it also carries a significant risk of access site complications, ranging from minor hematoma to major bleeding including hemoperitoneum. The use of hemostatic devices to suture the access site is more expensive. Furthermore, patients may experience discomfort, require exposure of their private parts, and need to lie down for a several hours. The transradial artery access (TRA) offers the advantage of reduced vascular complications and bleeding associated with vascular access (3). However, the most significant complication of the proximal radial approach is radial artery occlusion (RAO). The distal transdistal access (dTRA) has emerged as an alternative method to minimize occlusion.

This study investigates a novel bilateral distal radial artery strategy for subclavian artery stenting, addressing 3 critical gaps in current practice: (1) optimization of access site preservation in patients requiring repeated interventions, (2) development of cost-effective techniques through guide catheter elimination, and (3) establishment of bilateral access for subclavian artery lesion management. Moreover, it can be used in both CTO and non-CTO lesions.

### Cohort overview and indication

A total of 69 subclavian stent procedures were performed in our Cardiology Division 6, Xiamen Cardiovascular Hospital of Xiamen University, Xiamen, China, between 2021 and 2025. 30 cases were conducted via the radial approach (including both proximal and distal), 19 via femoral approach, and 10 cases utilized both radial and femoral approaches according to operator preference. Among these, we reviewed 10 cases of subclavian stenting procedures performed using a bilateral dTRA.

The median age of the patients was 65.80 (range: 54–79) years including 9 male and 1 female patient. 3 patients had hypertension, and other 3 had diabetes. 7 of them had high LDL cholesterol. 3 cases were asymptomatic and diagnosed with subclavian artery narrowing through color Doppler ultrasound. 6 patients presented with dizziness, while 1 presented with syncope and 1 presented with left hand numbness without neurological deficit. Dizziness was assessed using dizziness handicap index questionnaires (DHI). The systolic pressure difference between the left and right arms was 21.00 mmHg (range: 1–41 mmHg). 9 patients had coronary heart disease and 6 patients had cerebrovascular disease. All stent diameters were 8 mm except for patient 7 (7 mm). The median procedure time, was 23.10 minutes (range: 15–43 min). Patient baseline data and intraoperative details are shown in Tables 1 and 2.

**Table 1:** Patient characteristics.

Serial number	Sex	Age	HT	DM	Symptoms	Left pressure	Right pressure	IASBPD	LDL-C (mmol/L)	CHD/CAD
Patient 1	M	60	Yes	No	Dizziness (DHI-mild)	124/66	125/72	1	1.74	CHD
Patient 2	M	54	No	Yes	Nil	92/71	117/75	25	2.36	CVD
Patient 3	M	58	No	No	Dizziness (DHI-mild)	128/90	143/91	15	3.96	CHD,CVD
Patient 4	M	66	Yes	No	Nil	169/79	171/78	2	3.03	CHD,CVD
Patient 5	M	61	No	No	Syncope	92/60	123/70	31	1.15	CHD,CVD
Patient 6	M	69	Yes	No	Nil	122/77	152/82	30	0.74	CHD, CVD
Patient 7	F	79	No	No	Dizziness (DHI-moderate)	112/65	137/74	25	4.15	CHD
Patient 8	M	62	No	Yes	Dizziness (DHI-severe)	94/66	135/74	41	6.86	CHD
Patient 9	M	79	No	No	Dizziness (DHI-mild)	140/69	110/75	30	4.55	CHD, CVD
Patient 10	M	70	No	Yes	Dizziness (DI-moderate)	108/76	128/99	20	6.17	CHD

M, male; F, female; HT, Hypertension (Hypertension was diagnosed according to current hypertension guideline and patient was on antihypertensive treatment); DM, diabetes (diagnosed according to current diabetes guideline and patient was on antidiabetic treatment); DHI, (dizziness handicap inventory) score categorized as mild (0–30), Moderate (31–60) or severe (61–100); IASBPD, interarm systolic blood pressure difference; LDL-C, LDL cholesterol; CHD, coronary heart disease; CAD, cerebral cerebrovascular stenosis.

**Table 2:** Procedure, post-procedure of patients.

Serial number	Side	^a^Stenosis (%)	CTO	Sheath (F)	Angiography catheter	Predilatation balloon (mm)	Stent size (mm)	PT (min)	Contrast media consumption (ml)	Radiation dose (mGy)
Patient 1	Left	80	No	6	SM2	Aviator 5.0 × 20mm	8 × 37	15	60	400
Patient 2	Left	90	No	6	SM2	Aviator 5.0 × 30mm	8 × 27	15	70	380
Patient 3	Left	90	No	6	SM2	Nil	8 × 27	16	80	300
Patient 4	Left	80	No	6	SM2	Nil	8 × 27	20	90	330
Patient 5	Left	90	No	6	SM2	Nil	8 × 27	43	70	250
Patient 6	Left	100	No	6	SM2	Maverick 2.5 × 20mmAviator 5 × 30mm	8 × 17	34	60	220
Patient 7	Left	90	No	6	SM2	Aviator 5 × 30mm	7 × 27	23	100	350
Patient 8	Left	100	Yes	6	SM2	Maverick 3.0 × 20 mm Sterling5.0 × 30 mm	8 × 27	30	60	350
Patient 9	Right	90	No	6	SM2	Mustang 6.0 × 40 mm	8 × 27	20	50	280
Patient 10	Left	90	No	6	SM2	Aviator 5 × 30mm	8 × 27	15	80	370

^a^ Stenosis of the left subclavian was estimated visually with compared to angiographically normal segment, PT, procedure duration; Access site complications includes hematoma or bleeding, Aviator balloon (Cordis, Santa Clara, CA, USA), Maverick (Boston Scientisfic, Marlborough, MA, USA).

### Subclavian stenting procedure method of patient 8 (CTO subclavian stenosis)

**Figure 1:**
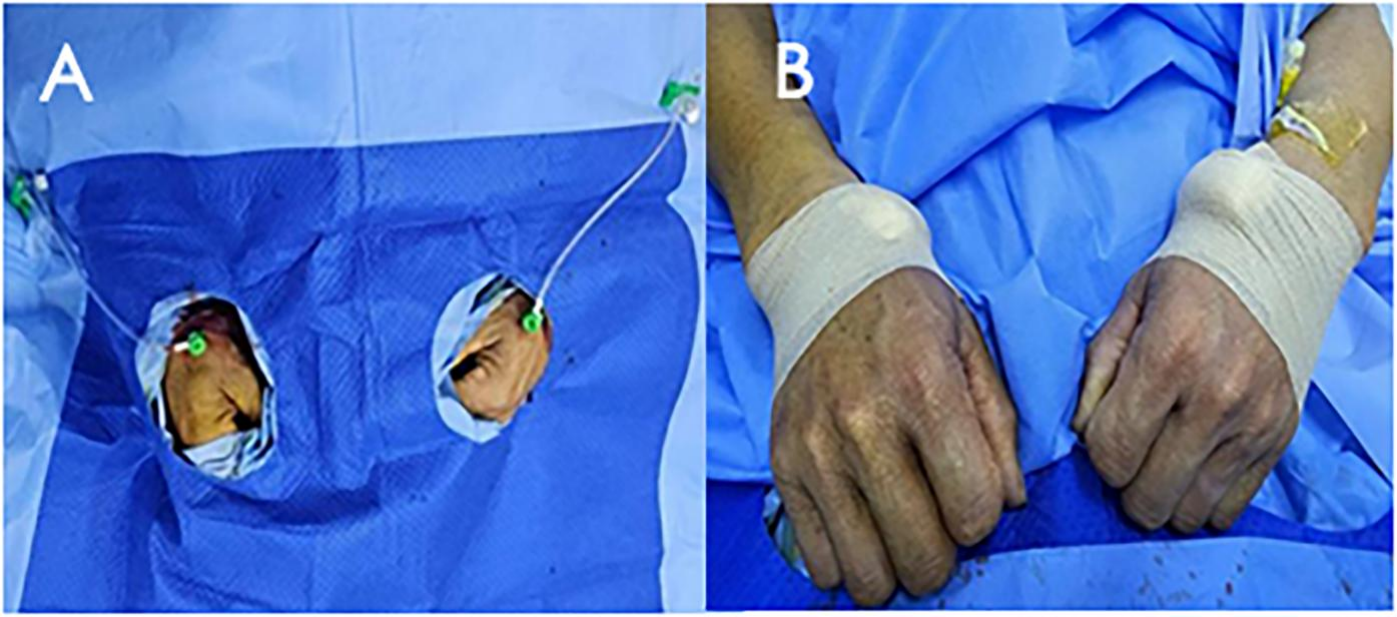
Bilateral distal radial puncture and inserted with 6F prelude merit sheaths **(A)** after procedure, haemostasis was secured with elastic bandage **(B)**.

The patient was informed about the procedural details, benefits, risks, and alternatives, and written consent was obtained. Under 2% subcutaneous lidocaine anesthesia, the right distal radial artery was punctured at the site of strongest pulsation over the trapezium or scaphoid bone, avoiding tendons (Figure 1A). Using the Seldinger technique, arterial access was obtained with a 6F Prelude Merit Sheath (Merit Medical Systems, South Jordan, UT, USA). The left distal radial artery was then accessed similarly using a 6F Prelude Merit sheath, which was ergonomically favorable for both the patient and operator (Figure 1B). The patient's hand was positioned in a neutral mid-position, with the anatomical snuffbox facing upward and the forearm slightly flexed over the patient's body. An unfractionated 100 units/kg of heparin were administered to maintain ACT between 250 and 300 s

**Figure 2:**
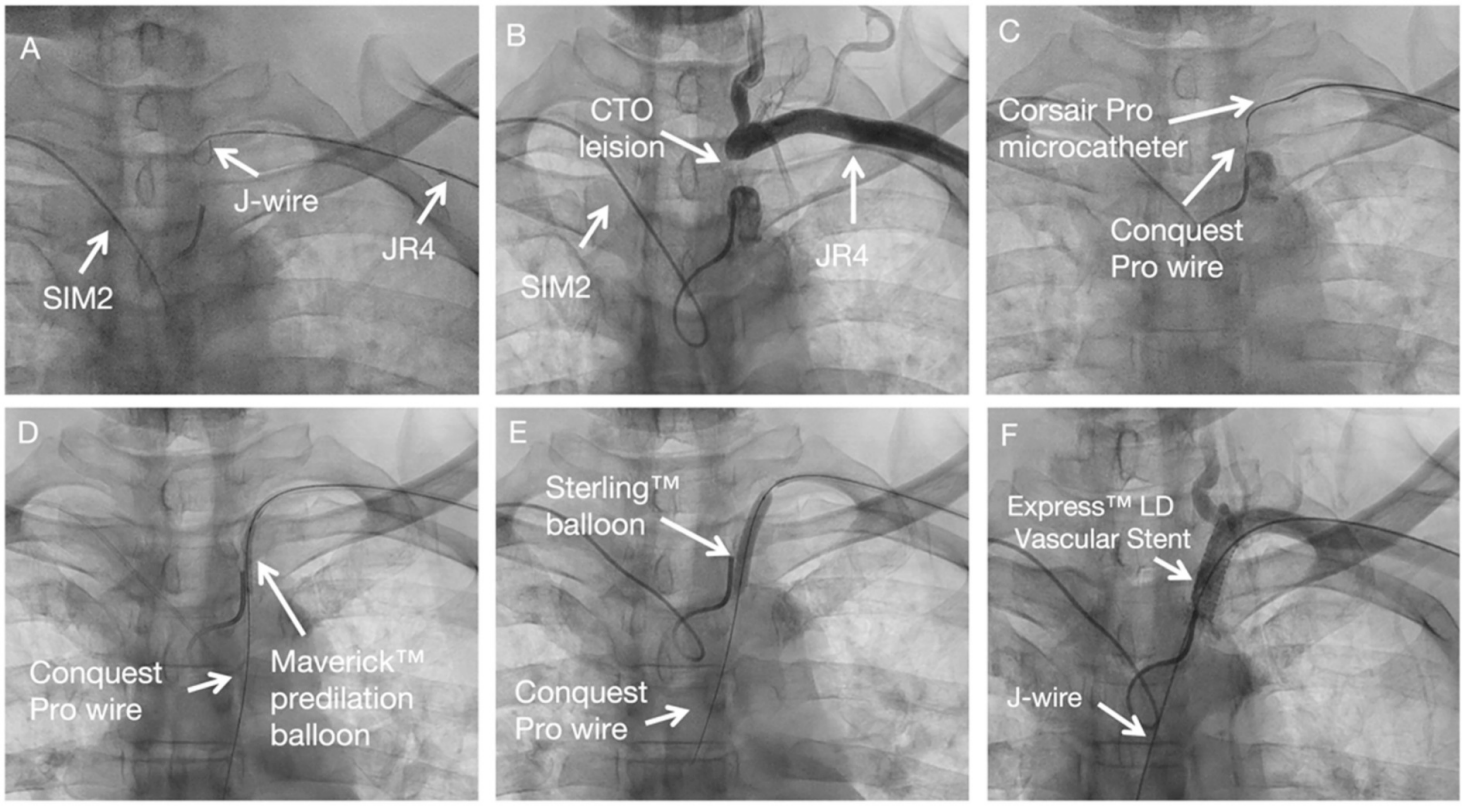
Subclavian stenting procedure method of CTO subclavian stenosis. **(A)** Diagnostic angiogram defining the occlusion. **(B–C)** Retrograde crossing of the lesion with a microcatheter and wire. **(D–E)** Sequential balloon angioplasty. **(F)** Final result after stent placement, showing no residual stenosis.

SIM 2 catheter from right-hand access engaged in the proximal cap of left subclavian artery stenosis and JR 4 advanced over J wire from the left hand access to the distal cap of left subclavian artery stenosis (Figure 2A). A diagnostic angiogram by SIM 2 catheter from right-hand access and JR 4 from left hand access showing proximal left subclavian artery stenosis total occlusion (Figure 2B). Corsair Pro microcatheter was used with a Conquest Pro wires to successfully cross the lesion retrogradely (Figure 2C). Contrast injection from SIM 2 to confirm the recanalization. Free movement of SIM 2 catheter over the Conquest Pro wire. Balloon angioplasty was performed with a Maverick™ 3.0 × 20 mm at 8 atm (Figure 2D). Balloon angioplasty was performed with a Sterling™ 5.0 × 30 mm balloon at 8 atm (Figure 2E). The Conquest Pro wire was then exchanged for a 260 cm J-wire from left hand access. An Express™ LD Vascular Stent 8.0 × 27 mm was delivered without a guide catheter and positioned across the lesion. The stent was carefully deployed at normal pressure (10 atm) without repositioning of the x-ray table to prevent displacement. The stent was fully expanded. Stent expansion, vessel wall apposition, and the absence of edge dissection were confirmed by angiography from SIM2 and showed no residual stenosis (Figure 2F). Technical details are available in the Supplementary Material 1.

### Subclavian stenting procedure method of patient 10 (non-CTO subclavian stenosis)

For non-CTO lesions, the same procedural steps were followed. A predilation balloon was advanced over the J-tip wire via left-hand access and was typically inflated at low pressure (8 atm). Subsequently, balloon-expandable stents were deployed over the J-tip wire without the use of guiding catheters.

**Figure 3:**
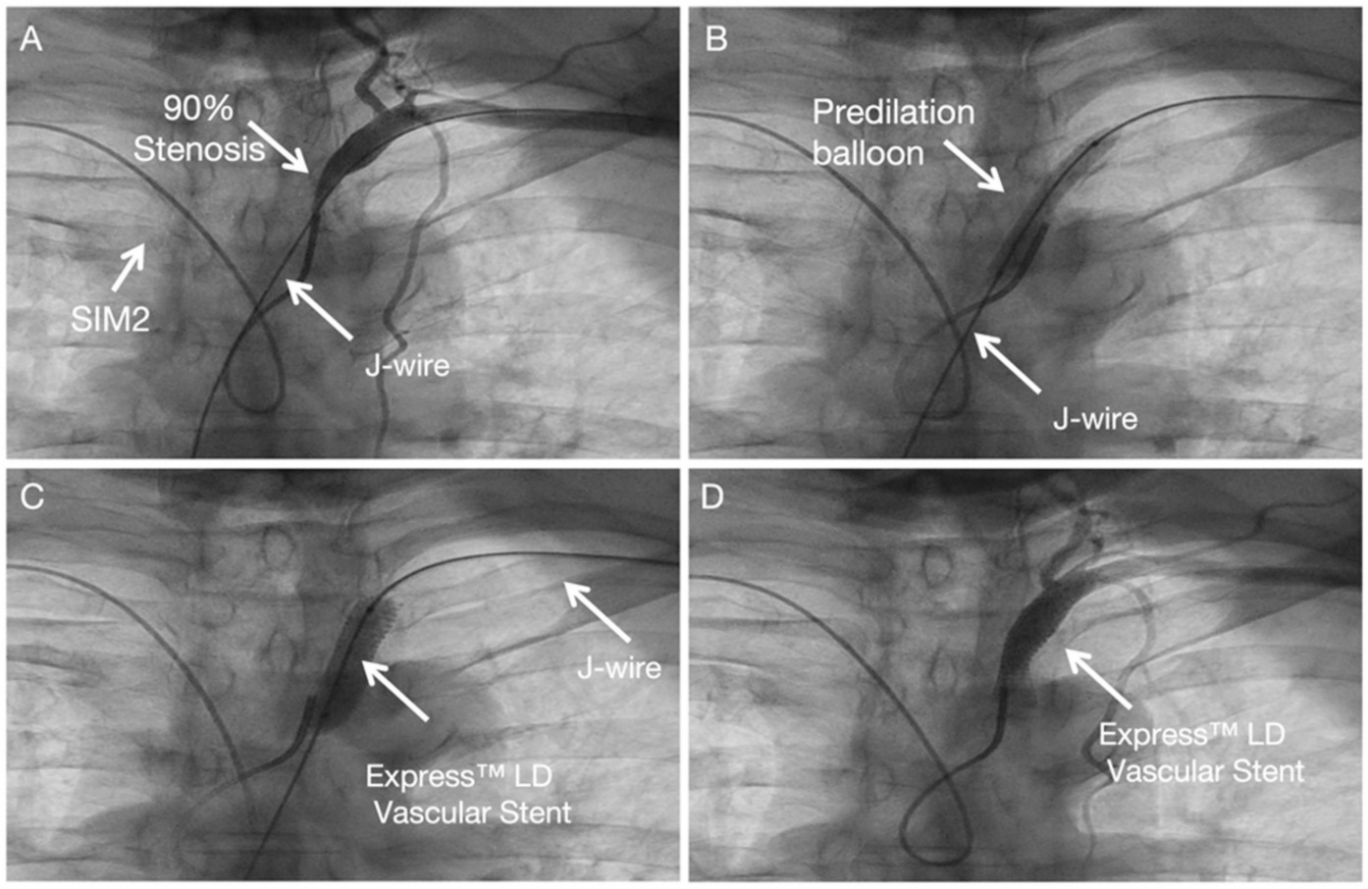
Subclavian stenting procedure method of left Non-CTO subclavian stenosis. **(A)** Engagement of the proximal lesion with a catheter from the right access and a wire from the left access. **(B)** Balloon angioplasty. **(C)** Stent deployment. **(D)** Final angiogram showing good stent expansion with only mild residual stenosis.

SIM 2 catheter from right-hand access engaged in the proximal cap of left subclavian artery stenosis and J wire from the left hand across left subclavian artery stenosis (Figure 3A). Balloon angioplasty was performed with a Aviator 5.0 × 15 mm at 8 atm (Figure 3B). An Express™ LD Vascular Stent 8.0 × 27 mm was delivered without a guide catheter, positioned across the lesion and deployed at normal pressure (10 atm) (Figure 3C). Stent expansion, vessel wall apposition, and the absence of edge dissection were confirmed by angiography from SIM2 and showed mild residual stenosis only (Figure 3D). Technical details are available in the Supplementary Materials 1, 2.

### Subclavian stenting procedure method of patient 9 (right subclavian stenosis)

The procedural steps mirror those for left subclavian artery intervention, with transposition of the angiography and intervention access sites. Technical details are available in the Supplementary Material 3, Figure 4.

**Figure 4:**
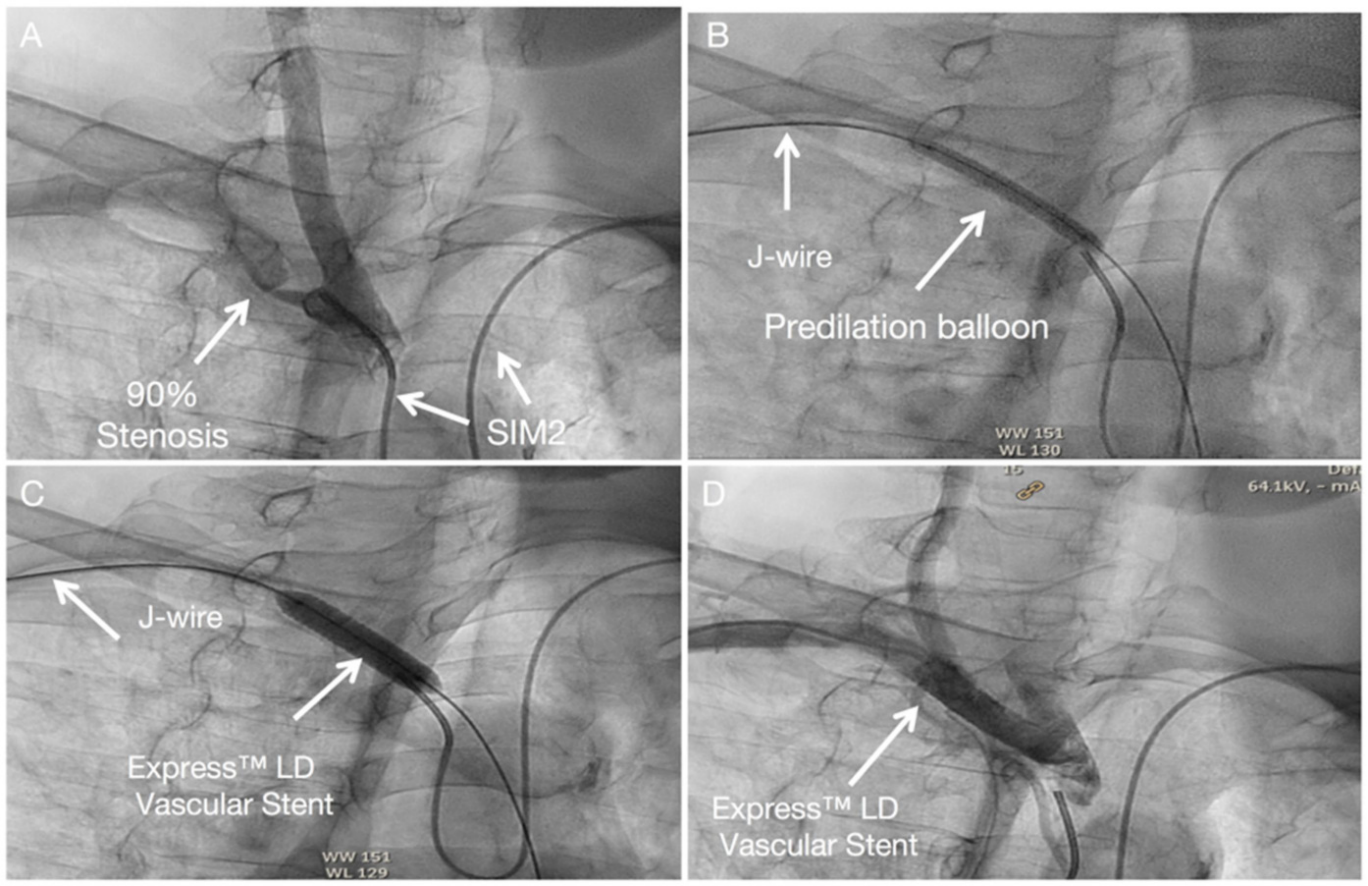
Subclavian stenting procedure method of right Non-CTO subclavian stenosis **(A)** SIM 2 catheter from left-hand access engaged in the proximal cap of left subclavian artery stenosis. **(B)** A predilation balloon was advanced over the J-tip wire via right-hand access and was typically inflated at low pressure. **(C)** An Express™ LD Vascular Stent was delivered without a guide catheter, positioned across the lesion and deployed. **(D)** Angiography from SIM2 showed no stenosis in stent.

### Postprocedural management

The sheath was removed immediately after the procedure and a single tourniquet was pulled tight over the puncture site. Post interventional medical treatment included aspirin 100 mg q.d. and clopidogrel 75 mg q.d. for 4 weeks followed by aspirin 100 mg q.d. No additional heparin was given after the procedure. Color-coded duplex sonography, interarm systolic blood pressure difference measurement were performed before hospital discharge and at 1-month follow-up and 1-year follow-up (case 9 was at 3-month follow-up).There were no access site complications, neurological complications or digital embolization. The average postoperative hospital stay was 1.3 days. The patency of both radial arteries was confirmed via pulsation at the wrist and snuffbox (Table 3).

**Table 3:** Post-procedure and follow-up data of patients.

Serial Number	Acces site hematomas	hemostasis time (h)	RAO 1 month	Neurological symptoms/signs	^a^Restenoss 1 month	^a^Restenosis 1 year	Postoperative length of hospital stay (d)
Patient 1	No	4	No	No	No	No	1
Patient 2	No	4.5	No	No	No	No	1
Patient 3	No	5	No	No	No	No	2
Patient 4	No	4	No	No	No	No	2
Patient 5	No	5	No	No	No	No	1
Patient 6	No	5.5	No	No	No	No	1
Patient 7	No	4	No	No	No	No	1
Patient 8	No	6	No	No	No	No	1
Patient 9	No	4	No	No	No	–^a^	1
Patient 10	No	5	No	No	No	No	2

^a^ Restenosis 1 month means significant narrowing of previous subclavian stents detected by color Doppler ultrasound postoperative 1 month; Restenosis means 1year means significant narrowing of previous subclavian stents detected by color Doppler ultrasound postoperative 1 year. Patient 9 had no restenosis at 3-month postoperative follow-up.

## Discussion

The “radial-first” policy has already been established in percutaneous coronary interventions (PCI) (4, 5). It is now also used in peripheral vascular procedures due to its lower invasiveness and high safety compared with TFA (6–9). Moreover, in the last few years, dTRA, a less invasive variant of TRA, has been introduced in PCI (10). In dTRA, the distal radial artery lies under the anatomical snuffbox, a triangular depression surrounded by the tendon of the extensor pollicis longus, extensor pollicis brevis, and the abductor pollicis longus, which is punctured (11, 12).The dTRA has emerged as an alternative technique aimed at reducing RAO rates and has been used in peripheral vascular intervention (13, 14). The risk of RAO is 2.92 times lower with dTRA compared to TRA, highlighting its potential benefits (3). As we know, patients with subclavian artery stenosis often present with concurrent stenosis in other arteries and may require multiple interventional procedures. Notably, among the 10 patients in our study, 9 were found to have coexisting coronary heart disease and 6 had cerebrovascular stenosis. Preserving radial artery patency can reserve access routes for subsequent interventional therapies (15). Importantly, none of the 10 patients in this study developed RAO, as confirmed by pulsation palpation at the wrist and anatomical snuffbox during the 1-month follow-up.

The use of a sheath with an outer diameter (OD) exceeding the inner diameter of the radial artery can lead to vascular wall stretch, endothelial injury, and chronic remodeling, thereby increasing the risk of occlusion (4). Given the higher RAO incidence associated with larger sheaths—11% for 6F (OD: 2.62–2.88 mm) and 19.5% for 7F (OD: 2.97–3.19 mm) (16)—this study utilized exclusively 6F sheaths to minimize RAO. For subclavian stenting, pre-dilation was performed using balloons measuring 5 mm in diameter and 20–40 mm in length. Balloon-expandable stents ranging from 7 to 8 mm in diameter and 15–20 mm in length were deployed. Delivering such large stents and balloons through a 6F guide can be challenging. To address this, the bilateral dTRA was used. For left CTO lesions, following retrograde lesion crossing via simultaneous catheter engagement (right-access SIM2, left-access JR4) and sequential balloon angioplasty, the stent was successfully delivered and deployed without a guide catheter. The bilateral dTRA also enabled successful subclavian artery recanalization for non-CTO lesions through simultaneous catheter engagement (contralateral-access SIM2, ipsilateral-access JR4), sequential balloon angioplasty, and guide catheter-free stent deployment via the ipsilateral-access. In this study, the bilateral dTRA offers significant procedural advantages in interventional therapy. Firstly, we avoid maneuvering guiding catheters within the aorta which significantly reduces the risks associated with guiding catheter use, such as aortic injury, dissection, or plaque disruption/embolization. Moreover, in patients with severely tortuous aorta, bilateral dTRA provides stable, coaxial alignment for enhanced support and trackability—improving device deliverability through challenging aortic anatomy. For subclavian artery CTO recanalization, bilateral dTRA is not just an alternative but a superior strategic choice. It provides the stable support needed to navigate tortuous anatomy, the built-in platform for retrograde techniques and precise angiography.

In our study, stents and balloons were delivered without a guide. The Express LD vascular stent, a premounted balloon system stent, was successfully delivered without a guide catheter. Accurate placement is critical, therefore, mapping with bony markers and structures from diagnostic angiography was employed. By maintaining the same x-ray view and table position, final confirmation was achieved using contralateral contrast injection via a SIM2 catheter. However, stenting without a guide requires expert operator skills due to the risk of stent misplacement and injury to adjacent structures. During the interventional process, we utilized PTCA wires, which provide excellent control and minimize the risk of vessel dissection. After achieving recanalization, the lesion was pre-dilated using a monorail balloon, followed by J-wire insertion for lesion preparation and stenting.

The omission of a guide and hemostatic devices, in comparison to the TFA, may resulted in a reduction in overall procedural costs for patients (8). Additionally, hemostasis time is shorter with distal radial artery, while overall bleeding and vascular complications do not differ significantly between the proximal and distal approaches (4). The mean time to hemostasis in this study was 4.78 hours. For TRA, patient positioning requires hand extension with the palm supinated over the body, which may be inconvenient, particularly for left-hand access. In contrast, dTRA allows for a more comfortable, neutral hand position.

dTRA has some disadvantages compared with conventional TRA. There are difficulties in arterial puncture and sheath insertion due to the small caliber of the vessel. Ultrasound-guided puncture, although requiring a learning curve, will also increase the success rate compared with palpation-guided puncture (17). However, the learning curve of dTRA is slighlty different and the use of larger sheaths (7–8 French) is currently being under investigation (18, 19). The most important aspect of this method is selecting a patient with good distal radial artery pulsation in the snuffbox. Second, arterial spasm was found to be more common in TRA compared to dTRA (20) Moreover, dTRA is generally considered to be more painful, possibly due to the risk of superficial branch radial nerve injury from repeated puncture attempts, leading to paresthesia and discomfort (21). Despite these concerns, our study did not observe significant arterial spasm or pain, nor did the overall procedure duration (23.10 ± 9.11 min) take longer.

Given the higher prevalence of left subclavian artery stenosis compared to right-sided lesions, our technique has been predominantly applied to left subclavian interventions. The procedural approach remains consistent for both sides: angiography is performed via contralateral access, while balloon angioplasty and stent implantation are delivered through ipsilateral access.

Limitations of this initial report include the small sample, limited follow-up duration, and absence of angiographic follow-up. A larger sample size cohort is required to evaluate whether dTRA intervention may fully replace the femoral approach.

Potential complications of subclavian stenting include dissection, distal digital embolization, and carotid embolization leading to infarction. However, carotid embolization is rare in subclavian stenting, and in our cases, cerebral protection devices were not used. Overall, there were no complications in our patients. Radial artery patency—a key study outcome—was assessed via pulse palpation at the wrist and snuffbox on postoperative day 1 and at 1-month follow-up. Although pulse weakening occurred in the proximal segment of 4 patients, patency was preserved without overt occlusion. Vascular remodeling may have occurred in these cases, warranting longer-term observation. To improve rigor, future studies will incorporate ultrasound confirmation and extended follow-up.

This study provides a novel and effective puncture strategy utilizing the bilateral dTRA for patients requiring interventional therapy for subclavian artery vascular diseases. Compared to traditional approaches via femoral or brachial artery access, this method facilitates faster patient recovery and reduces complications. It decreases intraoperative use of guide and hemostatic devices, thereby lowering medical costs. Relative to proximal radial artery access, it mitigates the risk of RAO, preserving vascular access for future interventions. However, this study has limitations: distal radial artery puncture poses challenges in patients with subclavian artery stenosis or occlusion, and may require blind puncture based on operator experience when arterial pulsation is weak. In conclusion, dTRA for interventional therapy in patients with subclavian artery stenosis is feasible. This puncture strategy offers clinicians a more minimally invasive approach to complete the procedure.

## Data Availability

The original contributions presented in the study are included in the article/Supplementary Material, further inquiries can be directed to the corresponding author.
